# Young Adults’ Perspectives on an Ecological Momentary Intervention for Drinking to Cope: Qualitative Study

**DOI:** 10.2196/85647

**Published:** 2026-06-25

**Authors:** Sarah E Dreyer-Oren, Oyindamola S Akinnusi, Bailey J O’Keeffe, Heather T Schatten, Michael D Stein, Zainab S Shah, Melissa Chery, Ana M Abrantes

**Affiliations:** 1 Department of Psychiatry and Human Behavior Brown University Providence, RI United States; 2 Butler Hospital Providence, RI United States; 3 School of Public Health Boston University Boston, MA United States

**Keywords:** anxiety, coping skills, depression, mobile apps, young adult

## Abstract

**Background:**

Young adults have high rates of mental health problems, such as mood or anxiety symptoms, and high rates of problematic drinking. Many young adults who undergo psychiatric hospitalization to address depression and anxiety symptoms also engage in risky drinking and tend to drink to cope with negative emotions. However, in many cases, treatment programs focusing on mood and anxiety symptoms often fail to adequately address problematic alcohol use in young adults.

**Objective:**

This study aimed to address this treatment gap by investigating patient perspectives on a potential ecological momentary intervention mobile app. Researchers used qualitative methods to gather perspectives of young adults hospitalized for psychiatric care on their use of drinking to cope with negative emotions and their feedback for a prospective app designed to suggest healthy coping strategies when participants report low mood and cravings to drink.

**Methods:**

We recruited a total of 12 young adults admitted to a partial hospitalization program to participate in a qualitative interview. To be eligible, participants needed to be aged 18-25 years and report drinking at least once weekly, binge drinking at least once monthly, drinking to cope with negative emotions, and depression and/or anxiety symptoms.

**Results:**

Qualitative analysis of our data resulted in 4 major themes. These included (1) motivations to use substances, (2) healthy coping, (3) general reactions to the proposed app, and (4) suggestions for the app. Participants generally had insight about their use of alcohol to cope and were able to identify several motivations for drinking; the most frequent motivations were to alleviate anxiety and depression, although many participants noted drinking to cope with other emotions, such as guilt or loneliness. Participants overall had positive responses to the prospective intervention and reported that they would appreciate the portability of a digital intervention in helping them “step down” from higher levels of psychiatric care. Participants also made several valuable suggestions about content, features, and usability, such as suggesting ways to “gamify” the app to increase use.

**Conclusions:**

This feedback will be crucial in designing and testing an ecological momentary intervention designed to reduce drinking to cope in young adults hospitalized for psychiatric care.

## Introduction

### Problematic Alcohol Use in Young Adults Hospitalized for Psychiatric Care

Young adults have high rates of mental health problems, such as mood or anxiety symptoms [1]. Young adults are also more likely than older adults to seek treatment and be psychiatrically hospitalized (ie, hospitalized primarily for mental health conditions such as mood or anxiety disorders). For example, compared with adults aged older than 25 years, young adults aged 18-25 years had higher rates of inpatient or residential treatment (2.1% vs 1.0% in 2023) [1] and higher rates of hospital-based outpatient treatment (1.5% vs 1.2%) [1].

Young adults also have high rates of problematic drinking. For example, in 2021, young adults were more likely than adults aged older than 25 years to binge drink (29.2% vs 22.4%) and more likely to meet criteria for alcohol use disorder (AUD) (15.0% vs 10.7%) [2]. Substance use disorders and other mental health disorders often co-occur, and this comorbidity is particularly common in young adults: 13.5% of adults aged 18-25 years had co-occurring substance use disorders and other mental illness in the past year, compared with 6.7% of adults aged older than 25 years [2]. Additionally, many people with significant mood or anxiety problems engage in hazardous drinking [3], including young adults who are psychiatrically hospitalized [4]. Young adults hospitalized for psychiatric care seeking treatment primarily for mood or anxiety disorders may engage in hazardous drinking and have significant negative drinking consequences, even if they do not meet criteria for AUD and/or do not desire to address alcohol use in treatment.

Many young adults who undergo psychiatric hospitalizations for internalizing problems, such as mood disorders, also engage in risky alcohol use and tend to drink to cope with negative emotions [4]. Drinking to cope (vs other motivations to drink, such as to enhance positive emotions) is associated with alcohol consumption and alcohol-related problems [5-7] and may be especially impactful for young adults [8]. Drinking to cope may augment the co-occurrence of mood and alcohol use problems because alcohol provides temporary relief from negative emotions, which in turn makes it challenging for people to cope with negative emotions more adaptively (eg, journaling or going for a walk). In addition to drinking exacerbating mood or anxiety problems by causing overreliance on alcohol to cope, problems with anxiety and mood may exacerbate problems with alcohol. Indeed, developing an anxiety or mood disorder is a risk factor for future development of AUD [9,10]. For these reasons, addressing problematic substance use in the context of general psychiatric treatment settings is imperative.

### Challenges for Treating Young Adults Hospitalized for Psychiatric Care With Co-Occurring Mood and Alcohol Use Problems

Problems associated with alcohol use can negatively affect young adults’ treatment. For example, substance use problems are associated with greater nonattendance of partial hospitalization programs [11]. Patients who are seeking treatment for internalizing problems may have low motivation to report substance use or related problems because of assumptions that addressing substance use in a treatment setting implies that their treatment goal should be abstinence from use. Indeed, not wanting to stop using substances is a frequently cited reason for not seeking substance use treatment [1], and thus, patients in primarily psychiatric settings may hesitate to report substance use.

Barriers within treatment settings also impact the treatment of co-occurring internalizing and alcohol problems. For example, one issue is the difficulty assessing and identifying substance use problems in general psychiatric settings. This underidentification may be due to providers’ lack of training in substance use disorders, stigma against substance use problems, or other factors [12] and can limit the effectiveness of treatment.

### Using Technology to Address Drinking to Cope

Because of these high mental health and alcohol-related risks for young adults, there is a pressing need to develop low-cost, scalable interventions for problematic drinking that take into account patterns of young adult drinking (eg, drinking to cope), as well as their preferences for treatment. Importantly, for people who believed they needed substance use treatment but did not receive it in 2021, cost-related concerns and not being ready to stop using alcohol were the most often reported barriers to accessing treatment (36.9% and 36.7%, respectively) [2]. Mobile interventions have much lower costs than other forms of mental health interventions, which helps mitigate cost as a treatment barrier. There are many mobile smartphone-based drinking reduction programs available, including some that show preliminary efficacy in reducing drinking; for example, one randomized controlled trial with participants discharging from residential substance treatment compared a smartphone app that combined self-monitoring and clinician monitoring with a treatment-as-usual control group and found that those in the intervention group had reduced drinking at follow-up [13]. However, meta-analyses of mobile interventions in general show mixed results [14-16], and adherence to digital treatments is relatively poor in general [17].

Additionally, focusing on interventions that are aimed at reducing problematic drinking patterns, such as drinking to cope, may also be more appealing than interventions that imply that sobriety is the goal, especially for young adults who are primarily seeking mental health treatment for mood and anxiety problems rather than substance problems. In line with the focus on problematic drinking patterns, to our knowledge, no mobile intervention exists that is focused on reducing drinking to cope specifically. For these reasons, it is imperative to hear perspectives from young adults with lived experience with drinking to cope in order to develop mobile interventions that are appealing, engaging, and effective. This aim aligns with a person-centered, formative framework for intervention development [18,19], in which potential users of proposed interventions are consulted during several stages of intervention development in order to maximize understanding of the intricacies and depths of users’ psychosocial environments.

### This Study

Using a qualitative design, this study aimed to investigate the perspectives of young adults hospitalized for psychiatric care on their use of drinking to cope with negative emotions, as well as their preferences for an app designed to suggest healthy coping strategies when participants report low mood and cravings to drink. This target population of the app would be young adults aged 18-25 years who engage in drinking to cope and are engaged in partial hospitalization psychiatric treatment, but are not primarily focusing on alcohol use in their treatment. In order to be eligible, participants needed to report drinking alcohol at least once per week, with at least 1 instance of binge drinking over the past month, depression and/or anxiety symptoms (as measured by depression and anxiety questionnaires), and drinking to cope with depression or anxiety (as measured by a drinking motives questionnaire). Note that participants were asked about their perspectives on the premise of a prospective app rather than their experiences using an app. The preliminary design of the app, based on past research conducted by this research team [20], was described to participants, who then gave feedback on their preferences for the app and methods that would increase usability and engagement.

## Methods

### Participants

A total of 12 participants (mean age 20.75, SD 1.91 years) were recruited from a young adult partial hospitalization program in a Northeastern psychiatric hospital. Participants were approached to assess for interest in and eligibility for the study if they met preliminary institutional review board–approved medical chart review criteria. To be eligible for the study, participants needed to meet the following inclusion criteria: (1) aged 18-25 years; (2) reported alcohol use at least once weekly over the past month and at least once monthly, on average, of binge drinking (defined as 4 or more drinks over 2 hours for those assigned male at birth and 3 or more drinks over 2 hours for those assigned female at birth); (3) self-reported use of alcohol to cope with negative emotions (mean score of 2 or more on the coping subscale of Modified Drinking Motives Questionnaire-Revised [MDMQ-R] [21], indicating they drink to cope at least “some of the time”); (4) current depression/anxiety symptomology (as assessed by Center for Epidemiological Studies Depression Scale [CES-D] [22] scores above the cutoff for high risk for clinical depression and Generalized Anxiety Disorder-7 (GAD-7) [23] scores above the cutoff for moderate to severe anxiety); and (5) ownership of a smartphone capable of downloading apps.

### Materials

#### Drinking to Cope

Drinking to cope with anxiety and depression was assessed using the coping with depression and coping with anxiety subscales of the MDMQ-R [21], an assessment adapted from the Drinking Motives Questionnaire-Revised [24]. The original 28-item MDMQ-R assesses 5 drinking motive subscales: coping with depression (eg, “to turn off negative thoughts about myself”), coping with anxiety (eg, “to reduce my anxiety”), conformity (eg, “to fit in with a group I like”), social (eg, “to be sociable”), and enhancement (eg, “because it’s exciting”), though only the 13 items assessing coping with depression and anxiety were included in this project. Items are rated on a scale from 1 (almost never/never) to 5 (almost always/always). Reliability scores for the coping with depression and coping with anxiety subscales were acceptable to good (Cronbach α=0.86 and 0.66, respectively).

#### Depression

The CES-D [22] was used to measure past-week depression symptoms. A total of 20 items (eg, “I felt sad”) are rated on a 4-point scale assessing frequency of symptoms, from 0 (rarely or none of the time [less than 1 day]) to 3 (most or all of the time [5-7 days]). Scores ≥16 indicate risk for clinically significant depression symptoms. Reliability in the current sample was good (Cronbach α=0.85).

#### Anxiety

The GAD-7 [23] measured the frequency of anxiety symptoms in the past 2 weeks. Items (eg, “not being able to stop or control worrying”) are rated on a 4-point scale from 0 (not at all) to 3 (nearly every day). Scores ≥10 indicate moderate anxiety, and scores ≥15 indicate severe anxiety. Reliability in the current sample was good (Cronbach α=0.88).

#### AUD Symptoms

The Alcohol Use Disorders Identification Test (AUDIT) [25] was used to measure AUD symptoms, including alcohol consumption and problems. Of the 10 items on the AUDIT, 8 items (eg, “How often during the last year have you found that you were not able to stop drinking once you had started?”) are rated on 5-point scales ranging from 0 to 4, with varying scale anchors, and 2 items (“Have you or someone else been injured because of your drinking?”) are rated on 3-point scales ranging from 0 (no) to 2 (yes, during the past year). AUDIT scores range from 0 to 40, with cutoff scores ≥8 for those assigned male at birth and ≥6 for those assigned female at birth, indicating hazardous drinking. Reliability in the current sample was adequate (Cronbach α based on standardized items =0.78).

### Procedure

Participants completed the screening questionnaire, which contained measures assessing drinking quantity, drinking to cope with depression and anxiety, and depression and anxiety symptoms. Patients who met eligibility requirements were asked to provide informed consent for participation in the audio-recorded interview. Participants then completed a questionnaire assessing sociodemographic information and AUD symptoms using the AUDIT.

Study clinicians conducted the qualitative interviews. They began with an overview of the interview process and then asked general questions about using alcohol to cope vs healthy coping. The study clinicians then explained the concept of the app, and explained that the app would send participants questions throughout the day and ask about their emotions and whether they intended to drink or were having cravings. If, based on the answers to those questions, it seemed likely that the participant would drink to cope with their emotions, the app would suggest several healthy coping strategies. Participants were not shown an actual app prototype; they were merely given a description of the app. After describing the proposed app, the interviewer asked several questions about participants’ thoughts about and suggestions for the app. Textboxes 1 and 2 provide a list of interview questions. The study clinicians then asked for any additional comments and concluded the interview.

**Qualitative interview questions about using alcohol to cope and healthy coping.**

**The interviewer described the premise of the study, which was to obtain the perspectives of young adults with anxiety and depression on the use of a smartphone app intervention to improve mental health and well-being.**

- “In general, in what ways do you think that drinking alcohol is related to unwanted or unpleasant emotions? How about anxiety and depression specifically?”
- “For you personally, does alcohol help you deal with unwanted feelings? Which emotions signal that you may want to drink soon? How effective do you think alcohol is in coping with these feelings?”
- “Besides drinking, what else helps you cope with your emotions? What are some healthier coping strategies or techniques you’ve used when you’re experiencing unwanted or unpleasant emotions?”
- “Why do you think you drink alcohol in some cases when you’re experiencing unwanted emotions, and use healthier coping options other times? What influences or determines this?”
- “Looking back, do you think you are aware when you’re having a strong unpleasant or unwanted emotion and using alcohol to cope with that emotion? To what extent are you aware of it at the time?”
- “Are there other coping strategies you have not tried before but think you may like to use in the future?”

**Qualitative interview questions about proposed intervention.**

**The interviewer described the proposed intervention smartphone app, which would ask users throughout the day to rate their negative emotions and indicate whether they plan to drink. Depending on users’ responses, the app will provide users with alternate, healthier coping strategies.**

- “What thoughts do you have about this general idea for helping you avoid drinking to cope with strong, unpleasant, or unwanted emotions?”
- “Which emotions should we ask about when determining whether to send suggestions for alternate coping strategies? On a 0-10 scale, where 0 is you’re not experiencing that emotion at all and 10 is the most intense you can imagine feeling that emotion, what do you think the rating would be that should prompt the app to send suggestions for coping strategies?”
- “How about urge rating level? At what point would your urge need to be on a scale from 1-10 to actually pursue drinking? Does it matter if it is available or not?”
- “To what extent do you think the alternate coping strategies should be individualized to your individual preferences versus a general list of strategies known to be helpful (based on prior research)? Or would you prefer both?”
- “For you, how many strategies would be useful to offer in any given instance?”
- “To what extent would you want to receive a follow-up text later in the day about whether: a) you used one of the alternate strategies and b) the extent to which using the strategy was helpful? If you would like this, would you want to be provided with additional strategies if it was not helpful?”
- “How many times during the day do you think is necessary to ask about your emotions to be able to capture a time when you may drink to cope? How many times a day would feel like too many times? Too few?”
- “What times during the day seem like the best times to send questions about your emotions and intention to drink alcohol?”
- “How would you prefer learning about strategies to avoid drinking? E.g., emojis or videos versus words/texts?”
- “What other features do you think would be helpful to include in a technology-supported intervention to help young adults receiving mental health treatment who use alcohol to cope with negative emotions develop healthier coping options?”
- “If you were to get messages daily for several weeks, how can we keep your interest?”

### Ethical Considerations

All study procedures were approved by the hospital institutional review board (application number 1735988). Participants signed an informed consent form after reviewing the form with the researcher and having the opportunity to ask questions. Participants were informed they could withdraw from the study at any time and had the option to decline to answer questions. Each participant was assigned an anonymized identification number, and identifying information was stored separately from study data and linked only by identification numbers. Interviews were recorded, and only designated and approved research staff had access to recorded data in order to transcribe interviews, which were free of identifying information. Participants were compensated with a US $30 gift card.

### Data Analysis

Thematic analysis [26] was used to analyze the interview data. Interviews were audio-recorded and transcribed by a study team member. Interviews were then coded for content and organized into themes. A total of 2 study team members developed an initial codebook, with deductive codes based on interview questions. A total of 6 study team members reviewed the initial codes and open coded 2 transcripts, adding inductive codes as needed to capture emergent concepts. The revised codebook was reviewed by the study team. Each remaining transcript, including the 2 previously open coded transcripts, was then independently coded by 2 raters. Rater pairs then met and assigned final codes via consensus. One author (SDO) reviewed all quotes and made suggestions for final coding at the end. Codes were entered by 2 study team members into NVivo (Lumivero) qualitative data analysis software. In this study, we present themes that emerged in interviews from at least 5 participants, which aligns with guidelines by Fugard and Potts [27] based on a sample size of 12, a 50% theme prevalence, and a power level of .80.

## Results

### Demographics and Descriptive Statistics

Participant demographics are displayed in Table 1. The sample was racially and ethnically homogenous. Participants held diverse gender identities and sexual orientations. The sample generally had high levels of internalizing symptoms: the mean depression score was in the severely depressed range (mean CES-D score 38.55, SD 10.01), and the mean anxiety score was just below the threshold for severe anxiety (mean GAD-7 score 14.17, SD 5.31). The sample also reported high levels of AUD symptoms, indicating a high likelihood of moderate or severe AUD (mean AUDIT score 15.25, SD 6.41).

**Table 1:** Demographic and descriptive data.

Variable			Frequency
Age (years), mean (SD)			20.75 (1.91)
Sex assigned at birth, n (%)			
	Female	8 (66.67)	
	Male	2 (16.67)	
	Other	1 (8.33)	
	Not Reported	1 (8.33)	
Gender identity, n (%)			
	Woman	5 (41.67)	
	Man	2 (16.67)	
	Trans woman	1 (8.33)	
	Gender nonconforming/nonbinary	4 (33.33)	
Sexual orientation, n (%)			
	Bisexual	3 (25)	
	Heterosexual/straight	3 (25)	
	Lesbian	1 (8.33)	
	Queer	1 (8.33)	
	Pansexual	2 (16.67)	
	Unsure/questioning	1 (8.33)	
	Other	1 (8.33)	
Ethnicity, n (%)			
	Not Hispanic or Latino/a	11 (91.67)	
	Not reported	1 (8.33)	
Race, n (%)			
	White or Caucasian	12 (100)	
Education, n (%)			
	Some high school	1 (8.33)	
	High school graduate/GED^a^	2 (16.67)	
	Some college	7 (58.33)	
	Bachelor’s degree	2 (16.67)	
Employment status, n (%)			
	Employed full-time	3 (25)	
	Employed part-time	3 (25)	
	Full-time student	1 (8.33)	
	Part-time student	1 (8.33)	
	Unemployed, seeking work	4 (33.33)	
Annual household income (US $)			
	0-25,000	3 (25)	
	25,000-50,000	4 (33.33)	
	75,000-100,000	2 (16.67)	
	100,000-125,000	2 (16.67)	
	>150,000	1 (8.33)	
Mental health measures, mean (SD)			
	Anxiety symptoms (GAD-7^b^)	14.17 (5.31)	
	Depression symptoms (CES-D^c^)	38.55 (10.01)	
	Alcohol use disorder symptoms (AUDIT^d^)	15.25 (6.41)	

^a^GED: General Educational Development.

^b^GAD-7: Generalized Anxiety Disorder-7.

^c^CES-D: Center for Epidemiological Studies Depression Scale.

^d^AUDIT: Alcohol Use Disorders Identification Test.

### Qualitative Themes

The qualitative analysis of our data, focused on understanding young adults’ healthy and unhealthy coping and their feedback on the description of the mobile app, resulted in 4 major themes. These included (1) motivations to use substances, (2) healthy coping, (3) general reactions to the proposed app, and (4) suggestions for the app. Some of these themes are organized into subthemes, detailed below.

### Theme 1: Motivations to Use Substances

#### Motivations to Use Alcohol and Use of Alcohol to Cope

Young adults expressed that they often use alcohol to cope with mood. In general, they discussed that alcohol provides relief from depression and anxiety. For example, participants noted that they drink to deal with “anger” and “guilt” (#1028); “anxiety, sadness, guilt, and shame” (#1031); “social anxiety” (#1040); “hopelessness,” “social anxiety,” “fear,” and “loneliness” (#1027); and “anxiousness,” and “worthlessness” (#1014).

Although almost all participants (11 of 12) reported that they used alcohol to ameliorate depression and/or anxiety, the most common mood-regulatory function described for alcohol was reduction of anxiety, particularly social anxiety (8 of 12 participants mentioned drinking to cope with stressors of social situations). For example, one participant noted that they drink to cope with “frustration, loneliness, maybe depression and definitely anxiety” (#1009). Although many participants acknowledged that drinking, in the short term, helped relieve depression, participants were more likely to acknowledge that drinking was effective in reducing anxiety vs depression. For example, a participant noted that drinking was more likely to ameliorate anxiety than depression, because alcohol is a depressant:

With anxiety, …[alcohol] loosens you up, makes you more confident…you lose your inhibitions, so you are not as worried…. with depression, [alcohol] is not as helpful because it brings you down… it does not serve as a distraction for depression.

#1014

Young adults also identified that drinking to cope with negative emotions was generally harmful, though it often worked in the short-term (10 of 12 participants acknowledged that alcohol was effective in coping sometimes but had long-term negative consequences, although 2 of those 10 noted mostly positive effects of drinking). Some participants acknowledged that drinking to cope with negative emotions was an indicator that their relationship with drinking was problematic. As one example of this idea, a participant said:

[Alcohol] is more like a Band-Aid, it’s not going to cure my depression or anxiety. It only covers it; it stops it from being present. It does not cure me and has negative effects long-term. I would call it unhealthy.

#1043

Another stated, “For me, drinking pushes emotions to the side for the moment, but almost heightens them after” (#1037).

In addition to helping cope with depression and anxiety, participants noted additional mood-regulatory functions of drinking. For example, some participants described alcohol as a “distraction,” “escape,” or “euphoria.” Several participants corroborated that they use alcohol to heighten their mood or escape from their day-to-day lives. For example, a participant stated:

From my experiences, people drink for the intoxicating high that it gives you and escapism. It makes people forget and it makes people lose their inhibitions.

#1043

A few participants also noted that they sometimes drink to deal with intrusive thoughts, such as obsessive thoughts or thoughts about past traumatic experiences.

#### Use of Substances Other Than Alcohol to Cope

Although the structured interview questions did not directly inquire about use of substances other than alcohol to cope, use of other substances (particularly cannabis) to cope with difficult emotions was an emerging theme in the interviews. Several participants expressed this pattern of using substances other than alcohol to cope when they were asked, “Besides drinking, what else helps you cope with your emotions?” A total of 7 participants reported using cannabis to cope with emotions, and 1 of those 7 also reported using psychedelic drugs to cope with emotions. Several participants reported co-use of cannabis and alcohol to cope with emotions.

### Theme 2: Healthy Coping

#### Overview

When participants were asked about what helps them cope with emotions, they noted numerous means of healthy coping. These coping strategies generally fell into several categories: distracting activities, grounding, movement and sensory activities, and activities to process thoughts and emotions. Participants also sometimes offered ideas for coping skills that they had not practiced but would like to try. Some participants also noted personal dislike of putatively healthy coping strategies that they had tried in the past.

#### Distracting Activities

Participants most frequently mentioned using distracting activities to cope. Some of these distraction-based coping skills were primarily recreational, such as word puzzles, watching television, playing video games or phone games, doing crafts, painting, reading, creative writing, listening to music, listening to podcasts, or playing musical instruments. Some participants also mentioned practicing productive distraction techniques, such as cleaning, organizing, or exercising. Several participants noted that spending time with others (eg, hanging out with friends or calling a friend) was a reliable coping skill. Several participants noted that the extent to which the coping skill captured their attention was critical in the coping skill’s usefulness. For example, one participant noted, “Music helps me a lot; I’ve played cello [to cope]. Especially improvised music is very helpful, because it is very ‘in the moment’” (#1027).

#### Grounding, Movement, and Sensory Activities

Some coping skills were centered on grounding participants in the present moment. For example, some participants also reported using movement-based coping strategies, such as exercising, practicing yoga, and walking. Participants also reported helpful sensory-based activities, such as exposing themselves to cold or hot temperatures (eg, submerging face in cold water), weighted blankets, or strong flavors (eg, eating a sour candy). Some participants also used breathing exercises and other mindfulness exercises (eg, progressive muscle relaxation) to connect to their internal physiological and sensory experiences.

#### Processing Thoughts and Emotions

Participants also reported that some coping skills help them by allowing them to interact with and process their thoughts and emotions. For example, journaling and reflecting on personal values were noted as helpful coping skills, and 2 participants recommended being able to write journal reflections within the app itself. Also, practicing acceptance was noted to be helpful by one participant:

[In treatment,] we have been learning about … accepting thoughts, feelings, and ourselves as we are... That can help with drinking, because lots of people drink because they do not accept … that is the biggest thing that I’ve been working through.

#1040

In particular, this participant noted that practicing acceptance was critical in changing their drinking.

#### Coping Skills to Try in the Future

Participants also noted coping skills they had not yet practiced but would like to try, often citing coping skills that were suggested to them during their treatment program. These include practicing mindfulness exercises, including guided meditation and breathing exercises, performing acts of kindness for others, and reading.

#### Personal Discomfort With Putatively Healthy Coping Strategies

Importantly, participants sometimes noted that certain categories of coping skills do not work well for them in certain scenarios; for example, one participant noted that grounding activities designed to connect them to their bodies and sensory experience were often recommended to them when they were young, and now they do not respond well when someone instructs them to do so. For example, a participant noted,

I had bad panic attacks when I was little.... I was frustrated by being told to breathe or calm down … [Being told these strategies] is triggering… the frustration of ‘that’s not helping.’ Now… I don’t even want to try it.

#1037

Like this participant, some participants suggested that users be able to decline or disallow certain coping skills from being suggested, which is discussed more in the section below about Theme 4 (app suggestions).

### Theme 3: General Reactions to the Proposed App

#### Overview

After discussing participants’ use of substances to cope and healthy coping, the researcher then described the proposed app to participants, explaining that the app would send participants questions throughout the day and ask about their emotions and whether they intend to drink or were having cravings. If, based on the answers to those questions, it seemed likely that the participant would drink to cope with their emotions, the app would suggest several healthy coping strategies. Participants were not shown a mock-up or prototype of the app; the interviewer simply described the proposed app. When asked about their general thoughts about the proposed app, participants overall had positive responses.

#### Positive Reactions to the Proposed App

All 12 participants generally had positive reactions to the proposed app. For example, one participant stated, “I think it’s a cool idea. I am glad somebody is concerned enough and has the resources to actually make it happen” (#1043). Another participant noted that the app might fill an important gap in their skill use:

[The idea of the app] is great, because I feel I never know what to do when I am feeling [negative emotions]. Like, what should I do right now other than drink? So that would be great, very helpful.

#1045

Some participants noted that several aspects of the app would be uniquely useful for their circumstances and the transition of stepping down from a higher level of treatment. For example, one participant noted that the proposed app might be helpful precisely because of the portability of a mobile phone app:

It’s definitely good to have coping skills [on a phone app], because I have made crisis cards around my house with coping skills, so I can think of them while I am in crisis, but my phone is always in my hands… [The app] would put [coping skills] in my head; I would not have to really think about what I could do.”

#1014

Participants also noted the app’s unique capacity to bridge care for patients discharging from time-intensive programs, such as partial hospitalization, to regular outpatient care, which allows for more unscheduled time. For example, one participant noted that the app would help them continue the practice of reflecting on their emotions, which they practiced in their partial hospitalization program:

[The app] is an amazing idea… It’s good to check in with yourself … especially leaving the program. I’m worried about not having someone to check in with like I did at partial… I’d like something to say, ‘how are you feeling?’

#1040

This participant also noted that having an external reminder of coping strategies could be especially helpful during times of stress:

Having alternative strategies come up for you [on the app] could be really helpful because, especially if you are in a space when you need [coping strategies], you might not really be thinking about them.

#1040

A total of 2 participants noted that the fact that the app uses technology rather than human interaction was a unique strength. For example, one participant stated:

[The proposed app] is good, especially because people get embarrassed talking about problems; I know I do. If there’s just an app, something you give what you’re honestly thinking, that is really helpful. I like the idea of ‘check-ups.’

#1028

Similarly, another said the relative anonymity of an app could aid in self-reflection:

People have problems being honest with themselves. But I feel if you’re honest with yourself enough to feel you need an app to monitor drinking, you should be honest enough to answer questions… it is just them and the app.”

#1018

These remarks highlight the particular benefits of using an app like the one proposed as a treatment adjunct.

#### Reservations About the App

Some participants had reservations about aspects of the intervention. For example, a participant noted the potential for the intervention to trigger symptoms:

The questions may be triggering... [users] might not be thinking of drinking at all and then the question comes up and reminds them. So, there should be information for helplines on [the app], for alcohol, the suicide hotline, and … telehealth.

#1037

Similarly, another participant noted that the app might not help them because substances are effective at reducing negative emotions, at least in the short term:

I worry, because at the end of the day I am drinking or smoking because I do not want to feel whatever I’m feeling. [Using substances] is the most effective way I found to not feel that.

#1027

This participant went on to say that the app questions may reinforce how they are feeling in the moment, which might be negative:

The app asking me how I am feeling, [which is] exactly what I am trying to avoid… the last thing I want to examine is my emotion in this present moment.

#1027

Nonetheless, the participant acknowledged that avoiding negative emotions was not as helpful as processing them, even though it was sometimes uncomfortable, and noted that their tendency to avoid emotions was harmful in the long-term, “which is why mindfulness can be so helpful” (#1027).

### Theme 4: Suggestions for the App

#### Overview

Participants made valuable suggestions about the app, both based on questions posed by the interviewers and spontaneously. In particular, participants made suggestions about the emotion-related questions, drinking-related questions, coping skills, delivery of feedback about coping skills, and strategies to increase usability and engagement.

#### Suggestions for Emotion-Related Questions

Several participants recommended that the app ask about depression and anxiety. Other common emotion suggestions were fear, sadness, loneliness, embarrassment, disappointment, shame, guilt, anger, worthlessness, panic, impulsiveness, and stress. A participant noted that it would be helpful to break down feelings like depression into different expressions of depression: “grief, guilt, regrets, low self-esteem” (#1043). Participants noted that personalizing the questions to match their specific symptoms would be helpful; for example, one participant with intrusive thoughts (#1045) stated that it would be helpful for the app to inquire about intrusive thoughts at each check-in. Another participant (#1037) suggested assessing for posttraumatic stress disorder symptoms. One participant (#1008) suggested that it would be important to assess happiness, though they did not explicitly state that happiness might trigger drinking for themself. Interestingly, one participant (#1040) suggested that the app ask about “emotional numbness” as an emotional state that would potentially trigger coping skill suggestions.

#### Suggestions for Questions About Cravings, Urges, and Intentions to Drink

When asked about phrasing of questions related to potential for drinking alcohol in the near future, such as questions about cravings, urges, and intentions to drink, participants generally expressed that it would be helpful to be able to answer multiple questions with different wordings. For example, a participant stated: “I think that would be best to [ask about] the urge and the intention” (#1040). They went on to explain that their answers to questions about urges vs intentions might be discrepant; for example, they might have strong urges to drink, but not have access to alcohol, and thus, offering coping strategies if either urges or intentions were endorsed would be useful. Another participant (#1037) suggested that participants be offered coping skills automatically if they endorsed urges or intentions to drink above a certain threshold, but to ask participants who were below that threshold whether they wanted suggestions for coping skills anyway, in case the urge to drink increased or was affected by triggers to drink, such as returning home after work. Some participants spoke about cravings and urges as equivalent to one another: “Thinking about [alcohol] and an overwhelming urge to use it… that would be pretty similar to craving it” (#1018). Another participant had a different perspective and distinguished between cravings vs urges:

It probably would be better to use “craving” than “urge,” because “urge” sounds very “addictive.” “Craving,” sounds almost happier; it does not trigger the sadness that comes with a word like “urges.”

#1042

#### Suggestions for Coping Skills

When asked about preferences for coping skill suggestions, generally, participants preferred to have some suggestions for coping skills that were new to them and some for coping skills that they knew worked well for them. “It should be a little bit of both: you can do some things you already like, and then new ideas” (#1045). Another participant noted that coping skill suggestions might be tailored to the situation and whether that skill tended to be effective for that situation in the past:

My coping strategies are situation-specific; if I found something that worked for that situation, I’d keep going with what works… but if it's someone who's newer in the process, they might like having variety so they don't feel locked in.

#1008

Thus, this participant acknowledged that offering a wide variety of skills rather than a few “tried and true” skills might be helpful for some users, but that they personally would rather be suggested skills they know are effective.

Several participants noted that it would be helpful to shape which coping skills were offered to them by providing information about which coping skills they were willing to use. “In the initial assessment, [it would be helpful] to be able to choose from coping skills, which ones apply to you or would be most helpful to you” (#1014). Some participants noted that there are some coping skills that were unlikely to be a good fit for them at any point (eg, “I know there [are coping skills] that, to me, they just do not really work. Like certainly meditation techniques, I cannot do a lot of that stuff” [#1040]). In addition to wanting to “opt out” of certain skills because they are unhelpful/unpleasant, participants noted that sometimes circumstantial constraints dictate how feasible certain coping skills are. For example, a participant said:

It’s important for strategies to be tailored to their habits [and] schedule... If somebody works two jobs and drinks to cope, they may not have time to go to the gym... something like writing down gratitude only takes a minute…

#1043

This participant suggested that asking participants what coping skills they have time for in their schedule and lifestyle would be valuable.

Participants also suggested that coping skills proposed by the app serve functions similar to the effects of alcohol. For example, one participant suggested*:*

It would be best to recommend [skills] that give a similar effect [to alcohol]. Because if you want the effects of drinking, and [the app] recommends [a skill] that doesn’t give those desired effects, you’re not likely to do it.

#1043

Likewise, several other participants had suggestions that centered on tailoring the coping skill suggestions to particular emotional contexts.

#### Suggestions for Coping Skill Feedback

Participants generally had a positive response to the idea of the app asking participants to give feedback about the coping skills that had been suggested, including whether or not they used the skill and the extent to which it was effective. Participants stated that this survey would be especially useful if responses were used to shape future coping suggestions.

A 15-minute check-in [after the coping skill suggestion is delivered] might be beneficial because you might be able to further personalize what coping skills are generated, and you can see trends on which ones work.

#1008

However, some participants were less enthusiastic about this prospect. For example, one participant surmised:

People would look at [a coping skill feedback survey] like they’re being nagged. Like, ‘hey, did you do that [skill]?’ And if people did it, it will make them not want to come back to the app and address it.

#1040

However, despite this reservation, overall, more participants favored the idea of giving coping skill feedback vs not giving feedback.

#### Features and Usability Suggestions

Several participants made suggestions for the app in terms of the interface and intervention delivery method. Participants suggested making sure there are visual and audio components to the app that make it more attention-grabbing and engaging, in order to compete with other things available on mobile phones, such as social media apps. Participants suggested using videos, emojis, quotes, or games within the app, for example, a virtual bubble-popping game, that could be incorporated in the app broadly or as part of coping skill suggestions. Several participants suggested incorporating a social element in the app. Another participant noted that the app’s use of language would affect the extent to which they stayed engaged in the app long-term and stated that it was important that the app communicate with a fun, nonjudgmental tone:

The specific language [of the app] is really, really important because I know my brain would be scanning for every little connotation. Like, ‘what does this app think about me and my substance use?’ and 'do I feel judged?’

#1027

In sum, these aesthetic- and presentation-oriented suggestions offered researchers ideas for how the app could maximize its appeal to users, both in the short and long term.

Some participants suggested ways to “gamify” the app and make it more interactive to increase its appeal. For example, a participant stated:

I think [you should] make [the app] like a game, because a lot of young adults are glued to their phones… play games on their phones, social media. So, do not make it look like a doctor would use it.

#1042

This gamification might enable the app to hold participants’ interest long-term; for example, another participant noted:

My age group tend to have a problem of attention span… Make it interactive, like getting a reward for carrying through for a certain amount of time; say, 2 out of the 6 weeks, you might get a coupon…

#1043

Altogether, several participants noted that incorporating game-like features, such as badges or achievements, would help hold their attention and support continued app use over time.

Participants also suggested that they would be more likely to use the app if there were a variety of ways to personalize it. For example, participants suggested that users be able to set the parameters of how many surveys delivered per day, when they arrive, and the thresholds of emotions, cravings/urges, and drinking intentions that would be required to prompt coping skill suggestions. Some participants also suggested personalizing the app by having it share participant trends and feedback, using data participants provide from answering surveys.

## Discussion

### Overview

In this study, which was designed to help understand the perspectives of young adults on drinking to cope and their preferences for an app designed to reduce drinking to cope, we interviewed 12 young adults. We asked them about their experiences with drinking to cope and their thoughts on healthy coping. We also described the prospective ecological momentary intervention (EMI) app and solicited their feedback about the premise of the intervention and ways to increase its utility.

The young adults generally had insight about their use of alcohol to cope and were able to identify several motivations for drinking. Anxiety and depression were the most common coping-based motives reported, though many participants noted other emotional states that were more complex and specific, such as guilt, loneliness, or emotional “numbness.” Several participants noted that drinking proved effective at relieving negative emotional states in the short term but acknowledged that in the long term, using drinking to cope was not effective and was sometimes harmful. This finding aligns with research suggesting that having coping motives for drinking was associated with subsequent negative affect [28].

The participants broadly had positive responses to the proposed intervention. They made several valuable suggestions about the questions assessing emotions and cravings, urges, intentions, and plans to drink. Furthermore, they had worthwhile insights into the app’s coping skill suggestions. For example, participants suggested that coping skills be tailored to the person and/or the particular situation they were in (eg, not suggesting a skill that was improbable or impossible to achieve). Many participants noted the value of distraction-based coping skills that took their minds off the negative emotions they were experiencing, such as creating art or playing a game. This aligns with a large meta-analysis indicating that distraction is an effective emotion regulation strategy [29]. Fewer participants mentioned coping skills that centered on processing or “sitting with” the emotions, although several participants did note that they practiced these skills (eg, journaling).

Other research has shown that there are several ways to personalize mobile interventions, including personalizing intervention content and personalizing through communicating with the user [30]. Participants suggested many ways to personalize the app, including when and how often to send surveys, what threshold of mood and drinking urges would warrant coping skills, and the content of the coping skills suggested. Several participants suggested having a self-monitoring component to the app, in which their responses would be aggregated to show them trends and data regarding their mood, alcohol use, and other behaviors. Although many participants noted that the app’s surveys would allow them to self-monitor their mood and alcohol use in the moment, they expressed wanting to see their responses over time. This suggestion aligns with several studies showing that self-monitoring is a popular behavior change strategy in mental health apps [31], and using such a strategy would align with other mental health–oriented apps available. Indeed, self-control–centered apps to reduce drinking, which tend to involve showing user progress over time, are generally more well-received by users than apps primarily focused on providing information through the app, such as daily messages or psychoeducation [32]. Based on this feedback, apps designed to help participants reduce drinking to cope should be designed to give users information about their own behavior over time in order to facilitate insight and motivation.

Our findings have several important clinical implications for our population of interest—young adults who are primarily targeting mood and/or anxiety symptoms in treatment and who also engage in drinking to cope. Because problematic substance use is frequently overlooked in primarily psychiatric treatment settings, incorporating the proposed app into clinical care could allow patients to increase their healthy coping skills and reduce the negative consequences of drinking to cope. Incorporating the app into clinical care could also help patients who are ambivalent about addressing drinking in treatment; because the app centers on reducing drinking to cope, rather than reducing or stopping drinking in general, patients who are less concerned with their drinking may still “buy-in” to the intervention.

### Limitations

Though this study had several strengths, including its use of a clinical sample, there are several limitations of this project to note. First, the sample was racially and ethnically homogenous, and only 2 of our participants were male, which limits the generalizability of our findings. Although this racial and ethnic homogeneity is not wholly unexpected given the small sample size and catchment area of the hospital from which we recruited participants (80.5% of the hospital’s catchment area population is White) [33], future research should emphasize gathering information from a more diverse sample to understand whether and how gender, racial, and ethnic identities shape understanding of ways to curb drinking to cope. Second, the interviews were framed around the interviewer describing the premise of a prospective app focused on suggesting coping skills to reduce drinking to cope, and thus asked relatively specific questions about participant preferences for such an app. Although this method is valuable in developing such an app, it may have proven fruitful to ask what participants thought would help reduce their drinking to cope more broadly, with less guidance about the prospective app from the interviewer.

While we focused on participants’ preferences and perceived benefits of an EMI intervention, we did not explicitly assess potential unintended consequences. Future research should explore possible negative effects, such as technology fatigue, excessive reliance on digital interventions, or potential distress caused by mood-tracking prompts, along with strategies for mitigating these effects. Finally, it would be helpful to recruit a sample from a wider array of treatment modalities, such as in traditional outpatient programs and/or inpatient programs, in order to increase study generalizability.

### Conclusion

To conclude, young adult participants in this study contributed valuable information about their use of alcohol to cope vs healthy coping strategies, as well as their responses to and recommendations for a prospective mobile app intended to reduce drinking to cope. This feedback will be crucial in designing and testing an EMI designed to reduce drinking to cope in young adults.

## Data Availability

The datasets analyzed during this study are not publicly available due to participant confidentiality concerns but are available from the corresponding author on reasonable request. Information about qualitative analysis methods and the study codebook is also available upon request.

## Abbreviations

AUD: alcohol use disorder
AUDIT: Alcohol Use Disorders Identification Test
CES-D: Center for Epidemiological Studies Depression Scale
EMI: ecological momentary intervention
GAD-7: Generalized Anxiety Disorder-7
MDMQ-R: Modified Drinking Motives Questionnaire-Revised
